# Composite Nanocoatings of Biomedical Magnesium Alloy Implants: Advantages, Mechanisms, and Design Strategies

**DOI:** 10.1002/advs.202300658

**Published:** 2023-04-25

**Authors:** Dan Li, Danni Dai, Gege Xiong, Shuquan Lan, Chao Zhang

**Affiliations:** ^1^ Stomatological Hospital School of Stomatology Southern Medical University Guangzhou 510280 China

**Keywords:** biological effects, composite nanocoatings, corrosion resistance, design strategies, magnesium alloy implants

## Abstract

The rapid degradation of magnesium (Mg) alloy implants erodes mechanical performance and interfacial bioactivity, thereby limiting their clinical utility. Surface modification is among the solutions to improve corrosion resistance and bioefficacy of Mg alloys. Novel composite coatings that incorporate nanostructures create new opportunities for their expanded use. Particle size dominance and impermeability may increase corrosion resistance and thereby prolong implant service time. Nanoparticles with specific biological effects may be released into the peri‐implant microenvironment during the degradation of coatings to promote healing. Composite nanocoatings provide nanoscale surfaces to promote cell adhesion and proliferation. Nanoparticles may activate cellular signaling pathways, while those with porous or core–shell structures may carry antibacterial or immunomodulatory drugs. Composite nanocoatings may promote vascular reendothelialization and osteogenesis, attenuate inflammation, and inhibit bacterial growth, thus increasing their applicability in complex clinical microenvironments such as those of atherosclerosis and open fractures. This review combines the physicochemical properties and biological efficiency of Mg‐based alloy biomedical implants to summarize the advantages of composite nanocoatings, analyzes their mechanisms of action, and proposes design and construction strategies, with the purpose of providing a reference for promoting the clinical application of Mg alloy implants and to further the design of nanocoatings.

## Graphical abstract

Composite nanocoatings can improve corrosion resistance and bioefficacy of biomedical magnesium alloy implants by the particle size dominance and impermeability of nanomaterials, and their specific biological effects and drug‐carrying by those with porous or core–shell structures. They can facilitate the application of magnesium alloys in the management of fractures, bone defects, atherosclerosis, esophageal or biliary stenosis, and other diseases.

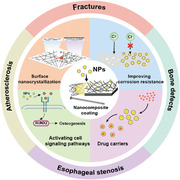

## 1 Introduction

Magnesium (Mg)‐based alloys have multiple biomedical applications due to their mechanical properties (including high specific strength and low density) that approximate those of human bone. Released Mg^2+^ ions are among the elements essential to cell division and proliferation, muscle contraction, and nerve conduction.^[^ ^1^, ^2^ ^]^ Unlike other biocompatible materials, biodegradable Mg‐based materials obviate secondary surgeries for implant removal, and thus reduce surgical trauma and foreign body reactions.^[^ ^3^, ^4^, ^5^ ^]^ However, the rapid degradation of Mg alloy erodes mechanical performance and may result in implant failure.^[^ ^6^, ^7^ ^]^ Surface modifications of Mg alloys may improve corrosion resistance and interfacial biocompatibility during contact with body fluids, and also serve as delivery vehicles for therapeutic agents. Thus, research concerning Mg alloy implants is focused not only on realizing controlled degradation and non‐toxic degradation products, but also on enhancing their biological activities.

Nanomaterial‐containing coatings provide a substrate with a nanoscale surface, controllable roughness, and hydrophilicity that facilitate cell adhesion and proliferation.^[^ ^8^ ^]^ They also exert antibacterial activity and enhance osteoblast differentiation and endothelial migration to accelerate tissue repair. Moreover, nanocoatings possess high‐density grain boundaries, interphase boundaries, and dislocations to improve corrosion resistance compared with traditional large‐particle coatings.^[^ ^9^, ^10^, ^11^ ^]^ In contrast to using nanoparticles (NPs) of monomaterials, the combination of multiple nano‐ or non‐nanomaterials to generate composite nanocoatings could exploit the unique and potentially complementary properties of different materials, thus enhancing biological effects in complex microenvironments. During the preparation of composite nanocoatings, blending schemes may improve toughness, reduce microporosity to prevent the penetration of corrosive biofluids, and regulate the degradation of Mg alloy to extend service time (**Table** **1**).^[^ ^12^, ^13^ ^]^ Furthermore, a superimposed or multilayer composite nanocoating can be prepared through a relatively easy preparation scheme to provide a multifunctional coating capable of promoting tissue repair and self‐healing, thereby improving the medical utility of Mg alloy (**Table** **2**).^[^ ^7^, ^13^ ^]^ However, the roles of blended or assembled composite nanocoatings, their interactions with other components, and their biological effects have not been summarized systematically.

**Table 1:** Nanomaterials (NMs) and their effects in mixed/doped nanocomposite coatings

NMs	Composite component	Substrate	Shape/size	Doping amount	Preparation method	Enhanced properties by NMs	Mechanism of the improved properties by NMs	Ref.
Ag NPs, Au NPs	PDA	AZ31	Ag NPs: 168.36 ± 48.5 nm, Au NPs: 151.03 ± 31.4 nm	–	Dipping	Anticorrosion, antibacterial	Reduced porosity; disturbed bacterial metabolism	[14]
CeO_2_ NPs	CS	Mg‐Ca	100–700 nm	1.0 wt%	Self‐assembly	Anticorrosion, self‐healing	Ce‐NH_2_ coordination for fixation; oxidized Mg to form a protective film; combined with OH^−^/H_2_O groups to produce insoluble Ce_2_O_3_ skeleton	[15]
CNTs, nHA	CS	AZ91D	–	CNTs: 0.5 g L^−1^; nHA: 5 g L^−1^	Electrodeposition (EPD)	Neurorestoration	Activated ERK/MAPK signaling pathway	[16]
COOH‐GNP	TEOS/MTES	AZ31	<4 nm	0.005, 0.05, 0.5, and 1 wt%	Dipping	Anticorrosion	Provided mechanical stability and compactness	[17]
curcumin/cFMSNs	DCPD/PLA/MgO	WE43	–	0.2 g L^−1^	Spinning	Anticorrosion, drug loading and sustained release	F^−^ passivates corrosion sites as a corrosion inhibitor; core–shell structure	[18]
Fe_3_O_4_ NPs	CS/BG	AZ91	–	1, 3, 5 wt%	EPD	Anticorrosion, osteogenic, antibacterial	Improved the dispersion and suspension stability of charged particles; Fe‐OH group induced heterogeneous nucleation of hydroxyapatite; Fe_3_O_4_ clusters produced micropores; penetrated bacterial cell membranes	[19]
GO, nHA	CS	AZ91D	GO sheet loaded foliated nHA, GO: 10 nm, nHA: <100 nm	0.5, 1, 2 wt%	EPD	Anticorrosion, antibacterial	Folding and rolling morphology; bridging and pulling mechanisms; mechanical properties and impermeability; sharp edges	[20]
HNT	PA, silk	Mg‐Ca	ID: 50 nm, OD: 15 nm	0.2% w/v	Electrospinning	Anticorrosion, self‐healing, osteogenesis	Loaded and released inhibiter PA; facilitate osteogenesis‐related gene expression	[21]
mSiNP	PLLA	WE43	Spherical	5, 10, 20 wt%	Spinning	Anticorrosion, re‐endothelialization	Increased densification and hydrophobicity; sustained released silicon ion	[22]
MWCNTs	AgSD/CS	Mg‐Zn‐Ca	274 ± 35 nm, 268 ± 28 nm, 254 ± 32 nm	0.25, 0.5, 1.5 wt%	Electrospinning	Anticorrosion, antibacterial	Increased densification	[23]
nHA	PDA/BMP‐2	AZ31	Needle‐like, long ≈ 120 nm, diameter ≈ 10 nm	0.2 g mL^−1^	Self‐assembly	Anticorrosion, osteogenesis	Increased densification; activated BMP signaling pathway; degraded to calcium and phosphorus ions	[24]
nHAC	PMMA/MgO	Mg‐Ca	–	15 wt%	Micro‐arc oxidation (MAO) + self‐assembly	Anticorrosion, osteogenesis	Reduced porosity; activated integrin *α*2*β*1‐FAK‐ERK 1/2 pathway	[25]
ZnO NPs	PCL, CM	AZ31	20–30 nm	0.25 mg mL^−1^	Electrospinning	Anticorrosion, osteoimmune	Reduced porosity; cleared excess ROS in macrophages	[26]

Ag, argentum; Au, aurum; BMP, bone morphogenetic protein; Ca, calcium; Ce, cerium; CeO_2_, cerium oxide; cFMSNs, F‐encapsulated mesoporous silica nanocontainers; CNTs, carbon nanotubes; —COOH, carboxyl; CS, chitosan; EPD, electrodeposition; F, fluorine; FAK, focal adhesion kinase; Fe_3_O_4_ NPs, ferroferric oxide nanoparticles; GO, graphene oxide; GOCS, chitosan‐functionalized graphene oxide; MAO, micro‐arc oxidation; Mg, magnesium; MgO, magnesium oxide; MTES, methyltriethoxysilane; nBG, nanobioactive glass; nHA, nanohydroxyapatite; —OH, oxhydryl; P, phosphorus; PCL, polycaprolactone; PDA, polydopamine; PLA, polylactic acid, PMTMS, polymethyltrimethoxysilane; TEOS, tetraethylorthosilicate; ZnO, zinc oxide

**Table 2:** NMs and their effects in layer‐by‐layer (LBL) assembly/superposition nanocomposite coatings

NMs	Composite component	Substrate	Shape/size	Doping amount	Preparation method	Enhanced properties by NMs	Mechanism of the improved properties by NMs	Ref.	
Cu@ZIF‐8, nHA	–	AZ31B	nHA: 367 nm, Cu@ZIF‐8: 1.646, 0.92, and 0.364 µm	–	Solvothermal	Anticorrosion, antibacterial, osteogenesis	Cluster Cu@ZIF‐8 particles filled the nHA; release Zn^2+^ and Cu^2+^ for antibacterial activity; Zn^2+^stimulated the MAPK pathway of rBMSCs, Cu^2+^ regulated the VEGF pathway of ECs	[27]
FeOOH	MgO	AZ31	Sheet‐like	4 g L^−1^	MAO+ self‐assembly	Anticorrosion, antibacterial	Reduced porosity; photocatalytic and photothermal activation	[28]
GO/MgAl‐LDH	MgO	AZ31	Sheet‐like	GO: 0.05, 0.1, 0.2 mg mL^−1^	MAO+ hydrothermal	Anticorrosion	Negative charge and specific surface area of GO	[29]
GOCS	Hep	AZ31B	0.8–1.2 nm	10 × 10^−3^ m	LBL self‐assembly	Anticorrosion, anticoagulation	—NH_2_ groups for fixation; —COOH groups inhibited platelet adhesion	[30]
MgF_2_	PDA	Mg‐Zn‐Y‐Nd	–	40% HF	Two‐step immersion	Anticorrosion, re‐endothelialization	Increased densification; altered hydrophilicity	[31]
MgF_2_	PDLLA	Mg‐Nd‐Zn‐Zr	–	30 wt% fluoride acid	Dipping	Anticorrosion, endothelialization	Endothelialized stent	[32]
Mg(OH)_2_	SA/PTMC	AZ31	Sheet‐like	–	Alkali treatment+ dipping	Anticorrosion, osteogenesis	Lamellar micro/nanostructure	[33]
Mg(OH)_2_/GO/nHA	–	Mg‐Ca‐Zn‐Ag	GO: flocculating sheet, nHA: sheet‐like	–	Hydrothermal+EPD+self‐assembly	Anticorrosion, antibacteria	Mg(OH)_2_ nanosheet blocked electron transfer; functional groups of GO chemically bonded to Mg(OH)_2_ and nHA; high hydrophilicity	[34]

BMSC, bone marrow mesenchymal stem cell; Ca, calcium; Cu, copper; Cu@ZIF‐8/HA, copper‐doped zeolitic imidazolate frameworks‐8 and inner hydroxyapatite; ECs, endothelial cells; EPD, electrodeposition; GO, graphene oxide; GOCS, chitosan‐functionalized graphene oxide; LBL, layer‐by‐layer; LDH, layered double hydroxide; MgO, magnesium oxide; Mg(OH)_2_, magnesium hydroxide; Mg, magnesium; Mg^2+^, magnesium ion; MgAl‐LDH, magnesium aluminum layered hydroxide; MgF_2_, magnesium fluoride; MgO, magnesium oxide; nHA, nanohydroxyapatite; PCL, polycaprolactone; PDA, polydopamine; PLA, polylactic acid; PMTMS, polymethyltrimethoxysilane; VEGF, vascular endothelial growth factor; Zn, zinc; ZnO, zinc oxide

Traditional surface modification schemes of Mg alloy primarily include chemical conversion (i.e., acid, alkali, and hydrothermal treatments; and sol‐gel coating); electrochemical methods (including electrodeposition and micro‐arc oxidation); physical deposition (such as dip coating, spin‐coating, electrospinning); and surface microstructural modification (pulsed electron beam and laser surface melting treatments).^[^ ^6^, ^35^ ^]^ These schemes can be used alone or in combination to prepare composite nanocoatings with nanoscale morphology or components on the Mg alloy surface, thus enhancing vascular regeneration, bone integration, and antibacterial activity, which expand the application range and improve therapeutic efficacy.^[^ ^36^, ^37^ ^]^ Therefore, this review summarizes the advantages of composite nanocoatings of Mg alloy implants that include mixed/doped and multilayer coatings prepared by layer‐by‐layer (LBL) assembly/superposition. Based on potential applications, we present an in‐depth analysis of the biological mechanisms of implanted nanomaterials, and propose design strategies for constructing Mg alloy, with the aim of providing a reference for promoting the clinical application of Mg alloy implants and furthering the design of medical nanocoatings.

## 2 Composite Nanocoating of Mg Alloy and Its Biomedical Advantages

**Figure 1:**
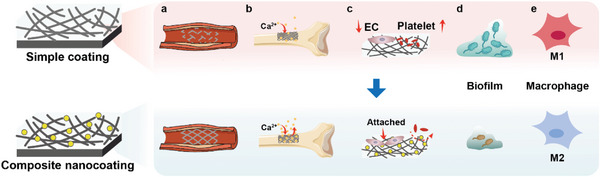
Advantages of composite nanocoatings on Mg alloy surfaces. a) Maintaining mechanical performance during service. b) Enhancing local hydroxyapatite formation and deposition. c) Promoting endothelial cell (EC) adhesion and inhibiting platelet adhesion. d) Destroying bacteria in biofilm. e) Inducing M2 macrophage polarization. Ca, calcium; ECs, endothelial cells.

Mg alloys used in biomedical implants are doped primarily with Zn, Ca, Y, and Zr, and have been used in the management of fractures, bone defects, atherosclerosis, esophageal or biliary stenosis, and other diseases. Mg alloy devices include fracture fixation devices (such as screws and bone plates), stents, and hemostatic clips.^[^ ^1^, ^4^ ^]^ Mg‐Ca, Mg‐Ca‐Zn, and AZ series (Mg‐Al‐Zn) Mg alloys are often applied in bone repair; while WE43 series (Mg‐Y‐rare element‐Zr), Mg‐Nd‐Zn‐Zr, and AZ31 Mg alloys are commonly used in cardiovascular stents. An implanted Mg alloy device must maintain sufficient mechanical strength during bone remodeling or revascularization, and may then degrade at a desired rate to meet treatment requirements.^[^ ^3^, ^6^ ^]^ On the other hand, degradation products should not provoke inflammation or predispose to infection, and should also promote cell adhesion, migration, and differentiation to confer therapeutic benefits (such as osseointegration and vascular reendothelialization) in the peri‐implant microenvironment.^[^ ^7^, ^35^, ^38^ ^]^ Most single‐component coatings cannot provide the full spectrum of sufficient mechanical performance, a suitable degradation rate, and ideal bioefficacy. The nanocomposite surface may combine the properties of multiple components to regulate the corrosion rate to maintain mechanical performance, and may also introduce bioactive nanoparticles to promote cell adhesion and colonization, reduce inflammation, inhibit bacterial growth, and provide a peri‐implant microenvironment conducive to tissue repair (**Figure** **1**).

### 2.1 Composite Nanocoatings Maintain the Mechanical Performance of Mg Alloy

The primary purpose of implants is to support tissue repair. The mechanical properties of Mg alloy should endure for at least three months for orthopedic implants and five months for vascular stents.^[^ ^6^, ^39^, ^40^ ^]^ However, the degradation rate of pure Mg and Mg alloy is much faster, thus jeopardizing sustained function. A pure Mg stent was completely absorbed in the coronary artery within two months,^[^ ^41^ ^]^ while rapid degradation of a Mg‐Zn‐Ca alloy rod was observed in the rabbit femur.^[^ ^42^ ^]^ Although magnesium oxide (MgO) ceramic coating^[^ ^12^ ^]^ and polymer coatings such as polycaprolactone,^[^ ^26^ ^]^ polydopamine,^[^ ^24^ ^]^ and aminated hydroxyethyl cellulose^[^ ^43^ ^]^ may raise the corrosion resistance of Mg alloys to a certain extent, their degradation rates are still too rapid for most clinical applications. A growing body of evidence suggests that the use of nanotechnology to modify the surfaces of Mg alloys may increase corrosion resistance. Modifications may include: i) the addition of nanoparticles, nanosheets, or nanofibers; ii) in situ formation of nanofilms [such as MgF_2_, Mg(OH)_2_] by acid or alkali treatments,^[^ ^31^, ^34^ ^]^ following the addition of polymer or ceramic particles to form composite coatings.

Mg alloys should meet varied mechanical requirements according to clinical applications. Orthopedic Mg alloy implants should immobilize displaced fractures, fill bone defects, and bear motive loads. Although a porous MgO coating of a pure Mg surface improved corrosion resistance 1.3‐fold, prevention of Cl^−^ penetration and subsequent local corrosion was still difficult. A rat calvarial defect model demonstrated delayed biodegradation of a Mg mesh cranioplasty insert that had been modified through plasma electrolytic oxidation and hydrothermal treatment to form a dense protective layer of MgO and calcium phosphate. CaP nanolayers on the MgO coating sealed surface pores, thereby preserving structure and confining degradation to a small focal area after 8 weeks, thus providing adequate mechanical support for bone healing.^[^ ^44^ ^]^ Mg alloy vascular stents must resist fatigue and fracture caused by pulse pressure and accelerated degradation induced by fluid shear stress, as biodegradation rates increase 3‐6‐fold under flow conditions.^[^ ^45^ ^]^ Vascular stents should also minimize the risk of thrombosis that may be provoked by localized corrosion and degradation products.^[^ ^46^, ^47^, ^48^ ^]^ A polydopamine (PDA) coating on a Mg‐Zn‐Y‐Nd alloy surface protected the substrate for only a short period (<6 d), and was degraded more than 80% after one month under the dual impacts of corrosive media and in vivo fluid dynamics.^[^ ^49^ ^]^ In contrast, a two‐layer coating consisting of a preprepared nanoscopic MgF_2_ film beneath PDA improved the barrier effect to repel corrosive media, and promoted tight bonding between the substrate and polymer that prevented the coating from stripping under fluid impact, and preserved mechanical performance longer than the unmodified PDA coating.^[^ ^31^ ^]^

### 2.2 Composite Nanocoatings Improve the Biological Properties of Mg Alloy

#### 2.2.1 Promoting Osteogenesis

In addition to permitting weight loading, Mg alloy orthopedic implants should promote the osteogenic differentiation of bone marrow mesenchymal stem cells (BMSCs) and the proliferation and migration of osteoblasts, and regulate the osteo‐immune microenvironment to stimulate callus formation and bone remodeling. Nanoscale surface topography provides an interface for extracellular matrix (ECM) adsorption that promotes cell adhesion and proliferation,^[^ ^50^, ^51^, ^52^ ^]^ while internal nanoparticles stimulate osteoblast differentiation and matrix mineralization.^[^ ^53^ ^]^ Osteogenesis at fracture sites may be accelerated by adding pro‐osteogenic nanoparticles with calcium, phosphorus, or silicon in coatings such as nanohydroxyapatite (nHA),^[^ ^54^ ^]^ nanobioactive glass (nBG),^[^ ^51^ ^]^ nano‐amorphous magnesium phosphate,^[^ ^55^ ^]^ and nano‐Ca_7_MgSi_4_O_16_.^[^ ^56^ ^]^ The use of metal oxide nanoparticles (such as Fe_3_O_4_ NPs)^[^ ^57^ ^]^ or adding oxhydryl (—OH)/carboxyl (—COOH) groups to the coating to provide crystal nuclei sites for mineralization are both effective.^[^ ^58^ ^]^ For example, the negatively charged Fe‐OH group of an Fe_3_O_4_ NPs/BG/chitosan (CS) composite coating induced heterogeneous apatite nucleation on an AZ91 Mg alloy surface. Micropores formed by coarse agglomeration of Fe_3_O_4_ NPs provided attachment points for the deposition of calcium and phosphorus ions, which accelerated the rates of apatite deposition and mineralization compared those of isolated BG coating, thus promoting bone healing.^[^ ^19^ ^]^ In addition, NPs added to composite coatings may also stimulate osteogenic or angiogenic signaling pathways to promote bone healing, as will be described in Sections 3.3.1 and 3.3.2 below.

#### 2.2.2 Preventing Vascular Stent Thrombosis and Restenosis

After intravascular placement, the lumenal surfaces of Mg alloy stents are absorbed by proteins (such as albumin, immunoglobulin, and fibrinogen) and stimulate platelet activation and adhesion, potentially initiating coagulation, stent thrombosis, and in‐stent stenosis. Superhydrophobic nanomaterials with low surface energy (such as diamond‐like carbon)^[^ ^59^ ^]^ inhibit adhesion; their addition may reduce platelet activation.^[^ ^60^ ^]^ A diamond‐like carbon/Cu composite nanocoating on an AZ31 Mg alloy surface served as a compact and uniform superhydrophobic film, which reduced platelet adhesion and maintained a low friction coefficient to prevent thrombosis, thus improving hemocompatibility.^[^ ^59^, ^61^ ^]^ Large surface areas of nanomaterials [e.g., graphene oxide (GO), multiwalled carbon nanotubes]^[^ ^30^, ^62^ ^]^ with abundant —COOH groups may enhance the anticoagulant activity of composite nanocoatings.^[^ ^63^ ^]^ A 16‐phosphonyl‐hexadecanoic acid/chitosan‐functionalized GO (GOCS) composite coating prepared on AZ31B Mg alloy decreased platelet adhesion (to ≈59% of that of bare metal) and activation, as evidenced by a 48% increased expression of cyclic guanosine monophosphate (cGMP), which reflects platelet inhibition. In addition, the composite nanocoating exhibited more potent anti‐platelet activity than the unmodified 16‐phosphonyl‐hexadecanoic acid coating; platelet adhesion was inhibited by 43% and the concentration of cGMP was increased by 22%.^[^ ^30^ ^]^

The endothelialization of vascular scaffolds and proliferation of vascular smooth muscle cells (SMCs) also affect Mg alloy stent efficacy. With the degradation of Mg surfaces, endothelial cells (ECs) and SMCs are gradually exposed to relatively high local Mg^2+^ concentrations that upregulate gene expressions related to angiogenesis and cell adhesion signaling pathways to promote migration and proliferation. Although endothelialization supports stent function, SMC proliferation initiates vascular “negative remodeling.” Foreign‐body‐mediated inflammation and chronic vascular distention induced by Mg alloy scaffolds may accelerate the migration and proliferation of SMCs and cause restenosis. Thus, the chemotactic differences between SMCs and ECs on specific nanomaterial‐modified surfaces may be exploited to regulate cell adhesion selectively. This approach may improve the proliferative advantage of ECs over SMCs and inhibit SMC proliferation to promote stent endothelialization.^[^ ^64^, ^65^ ^]^ A directly‐deposited PDA coating on a Mg‐Zn‐Y‐Nd alloy was highly hydrophilic [water contact angle (WCA) = 80.3 ± 0.7°], and promoted SMC proliferation to twice the rate of ECs.^[^ ^66^ ^]^ In contrast, MgF_2_ nanoparticles reduced WCA to 28.01 ± 2.98°, which exerted the opposite effect; EC proliferation was twice that of SMCs. Enhanced EC proliferation accelerated endothelialization and inhibited maladaptive proliferation and growth of SMCs, thereby preventing in‐stent stenosis and tissue scarification.^[^ ^31^ ^]^

#### 2.2.3 Anti‐Infective and Immunoregulatory Activity

Infections of open fractures and orthopedic implants impede osteointegration. Bacteria may escape host immune responses and upregulate the expression of osteoclastogenic cytokines, causing pathological bone loss and delayed healing.^[^ ^67^ ^]^ Inflammatory cell infiltration leads to osteolysis, medullary cavity expansion, abnormal bone thickening, and deformed bone.^[^ ^68^ ^]^ Thus, Mg alloy implants in contaminated and chronically inflamed sites must exert antibacterial activity (such as tissue disinfection and inhibition of bacterial adhesion and proliferation) and immunomodulatory properties to promote bone healing.^[^ ^69^ ^]^

**Figure 2:**
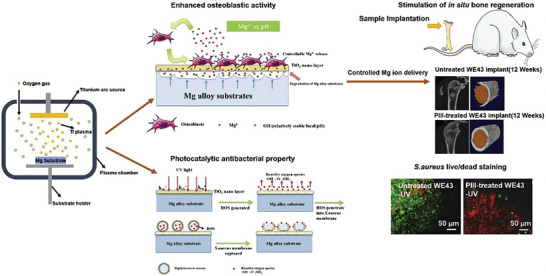
TiO_2_/Mg_2_TiO_4_ nanolayer prepared on WE43 Mg alloy enhances osteoblast function and exerts photocatalytic antibacterial activity to stimulate in situ bone regeneration. Reproduced with permission.^[^ ^78^ ^]^ Copyright 2019, American Chemical Society.

Bactericidal nanomaterials (such as boron nitrides; 2D graphene and its derivatives;^[^ ^70^, ^71^ ^]^ metal nanoparticles such as Ag, Au, and Cu; and metal oxide nanoparticles including ZnO, TiO_2_, and Fe_3_O_4_)^[^ ^14^, ^19^, ^23^, ^27^, ^72^, ^73^, ^74^ ^]^ act by altering membrane permeability or by catalyzing reactive oxygen species (ROS) to initiate oxidative stress. The 2D structures of boron nitride nanosheets embedded in chitosan feature sharp edges that rupture bacterial cell membranes, thus impeding bacterial adhesion and biofilm formation, and thereby prevent pathological bone loss.^[^ ^51^ ^]^ After implantation into an infected rat femoral condyle defect model for four weeks, a Mg‐Ca‐Zn‐Ag alloy with a Mg(OH)_2_/GO/HA composite nanocoating inhibited bacterial proliferation, reduced inflammatory cell infiltration, inhibited osteolysis, and promoted bone regeneration.^[^ ^34^ ^]^ Ag and Au nanoparticles incorporated into a PDA coating on an AZ31 Mg alloy killed more than 50% of *S. aureus* and *E. coli* within 24 h and enhanced bone remodeling.^[^ ^14^ ^]^ Copper‐doped zeolitic imidazolate frameworks‐8 and inner hydroxyapatite (Cu@ZIF‐8/HA) NPs grown in situ on the micro‐nano flower‐like structure of nHA exhibited sustained release of Cu^2+^ and Zn^2+^ to achieve 99.99% bacterial killing, and also enhanced in vitro osteoblast activity.^[^ ^27^ ^]^ Nanomaterials with stimulus‐responsive catalytic activity (e.g., TiO_2_ and FeOOH) may generate ROS under exogenous stimulation (such as light),^[^ ^28^, ^75^ ^]^ thus killing bacteria non‐invasively and avoiding the risks of antibiotic therapy that include toxicity, hypersensitivity, and antimicrobial resistance. The advantages of this technique include its outstanding controllability and targetability. Regulating the particle size of nanomaterials and the intensity and duration of external stimulation may modulate bactericidal activity and reduce secondary infections.^[^ ^76^, ^77^ ^]^ A TiO_2_/Mg_2_TiO_4_ nanolayer prepared on a WE43 Mg implant reduced oxidative stress of adjacent tissue and cells while killing methicillin‐resistant *S. aureus* through ROS production induced by ultraviolet irradiation, suggesting that such alloys could treat multi‐drug‐resistant bacterial infections without injuring host tissue (**Figure** **2**).^[^ ^78^, ^79^ ^]^

Immunomodulatory nanomaterials in composite coatings may reduce inflammation and promote tissue repair. For example, *β*‐tricalcium phosphate (*β*‐TCP) may inhibit toll‐like receptor signaling to promote M2 macrophage polarization, which suppresses local inflammation and osteoclast differentiation. *β*‐TCP also activates the BMP‐2 signaling pathway of BMSC to enhance osteoblast differentiation.^[^ ^80^ ^]^ A CaHPO_4_ and *β*‐TCP composite nanocoating grown on an AZ31 Mg alloy not only supplied calcium and phosphorus, but also induced macrophage polarization to the M2 phenotype to facilitate an adaptive osteoimmune microenvironment, thus promoting bone healing. Lanthanum‐substituted MgAl‐layered double hydroxide (La‐LDH) may neutralize the acidic microenvironment of osteoporosis to reduce osteoclast activity and increase M2 macrophage polarization.^[^ ^81^, ^82^ ^]^ A composite nanocoating comprised of La‐LDH with GO may regulate osteoclastogenesis and osteogenesis simultaneously, which would be of exceptional value in the treatment of osteoporosis‐related fractures.^[^ ^29^, ^83^ ^]^ In addition, nanomaterials can serve as microcontainers to load immunomodulatory drugs (e.g., curcumin, statins),^[^ ^18^, ^84^ ^]^ which stimulate reparative M2 macrophage polarization to improve the osteoimmune microenvironment and reduce inflammation. F‐encapsulated mesoporous silica nanocontainers (cFMSNs) loaded with curcumin have been dispersed in a poly(l‐lactide) interlayer to modify Mg alloy implants for craniofacial bone reconstruction. These NPs enable a biphasic drug elution that features an initial high‐dose delivery followed by a stable release. This pharmacokinetic profile promotes the transformation of M2 macrophages, creates an osteogenic microenvironment, promotes the osteoblastic differentiation of BMSCs and mineralization of the extracellular matrix, thereby accelerating bone fusion.^[^ ^18^ ^]^

In addition, Mg alloys serving as nerve guide conduits or scaffolds can provide structural support to guide axon regeneration for large nerve defect treatment. Nanomaterial‐modified surfaces of Mg alloy can support cell attachment, morphology, and arrangement, and the deposition of bioactive nanoparticles could promote the proliferation and migration of neurons.^[^ ^85^, ^86^, ^87^ ^]^ Bare magnesium filaments were excellent conductors to enhance the electrical stimulation or signal transduction of neurons, and the release of Mg^2+^ could promote the repair of peripheral nerve and spinal cord injury.^[^ ^88^, ^89^ ^]^ The addition of electroconductive and neural inducing nanomaterials (such as carbon nanotubes and black phosphorus)^[^ ^16^, ^90^ ^]^ into the coating can further facilitate the axon extension and neuroregeneration, and the specific mechanism was described in Section 3.3.4 below.

## 3 Mechanisms of Composite Nanocoatings to Improve the Utility of Mg Alloy

### 3.1 Surface Nanocrystallization Improves Surface Properties and Cell Affinity

Cell–implant surface interactions involve ECM protein deposition, cytoadhesive dynamics, and cell migration. Almost all cell types respond to nanoscale surface properties that include topography, hydrophilicity, and surface charge. These characteristics promote protein adsorption; filamentous pseudopod formation; and cell adhesion, proliferation, and differentiation.^[^ ^91^, ^92^ ^]^ Composite nanocoatings endow Mg alloy nanoscale surfaces with surface topographies that facilitate the adsorption of adhesive proteins and subsequent selective cell adhesion and proliferation.^[^ ^93^, ^94^ ^]^ First, nanotopography aligns actin along the direction of cues, elongates cellular pseudopodia toward nanocues (such as nanogrooves), and induces cell migration to generate tissues. Compared with flake‐like micro‐hydroxyapatite, rod‐like nano‐hydroxyapatite deposited on a Mg‐Zn‐Ca alloy coated with MgO arranged neatly in a consistent direction promotes MSC adhesion and migration on implants.^[^ ^95^ ^]^ In addition, nanoscale surfaces guide the assembly of integrin, a cell adhesion protein aggregate that facilitates the synthesis of intracellular tight junctions and promotes the adhesion and osteoblastic differentiation of BMSCs.^[^ ^96^ ^]^ A double‐layer phytic acid@cerium/polycaprolactone (PCL)@cerium‐substituted hydroxyapatite composite nanocoating with a high aspect ratio of nanofiber structures on an AZ31 Mg alloy provided an ECM‐like interface, which induced BMSCs to express endogenous hyalin to link integrin clusters and form larger and more mature pseudopods on the nanosurface, thus promoting cellular proliferation and differentiation.^[^ ^97^, ^98^ ^]^

In addition to morphology, other physicochemical properties such as surface charge and hydrophilicity affect responses at the material–cell interface at the molecular (e.g., protein adsorption) and cellular (e.g., as differentiation, proliferation, and migration) levels.^[^ ^99^ ^]^ These characteristics regulate the strength and degree of cell adhesion mainly by interacting with cell surface adhesion receptors (e.g., integrins), thereby influencing proliferation and the switch between proliferation and differentiation.^[^ ^91^ ^]^ Positively charged nanosurfaces enhance cell adhesion because ECM molecules are negatively charged and consequently better adsorbed to positively charged implant surfaces to form spatial conformations that favor cell adhesion and differentiation.^[^ ^91^, ^100^ ^]^ For example, positively charged GOCS and heparin were deposited on an AZ31B Mg alloy surface by LBL self‐assembly.^[^ ^30^ ^]^ The outermost GOCS layer increased local concentrations of collagen, lamin, and fibulin, promoting EC adhesion and consequent reendothelialization.^[^ ^30^, ^101^ ^]^ In general, implant WCAs in the range of 35°–80° are more favorable for cell adhesion.^[^ ^102^ ^]^ Adding granular nanomaterials (such as nBG, Ce‐HA NPs, and SiO_2_ NPs) in the composite coating creates a surface with nanoscale roughness that enhances hydrophilicity.^[^ ^51^, ^97^, ^103^ ^]^ Doping nBG into PCL coatings reduced WCA from 119 ± 7° to 69 ± 6°, thus promoting osteoblast adhesion and proliferation.^[^ ^104^ ^]^

### 3.2 Improving Corrosion Resistance to Control the Degradation Rate of Mg Alloys

**Figure 3:**
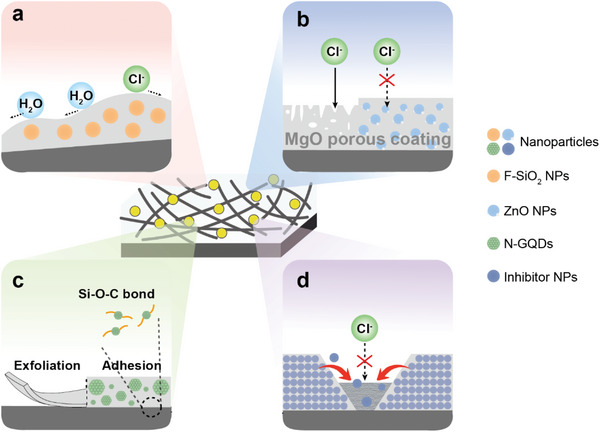
Composite nanocoatings improve corrosion resistance to control the degradation rate of Mg alloy. a) Insulating layers resist the penetration of corrosive fluids. b) Reduced porosity decreases the degradation rate. c) Enhanced adhesion of the coating and substrate enhances bond strength. d) Nanomaterials in coatings may inhibit corrosion by passivating the substrate or by generating degradation products that cover the substrate, thus exerting a self‐healing effect.

Composite nanocoatings may serve as physical barriers to improve the corrosion resistance of Mg alloy implants. First, the low permeability of 2D nanomaterials (e.g., graphene and its derivatives, and LDH) reduces alloy exposures to corrosive fluids (**Figure** **3**a).^[^ ^29^, ^105^ ^]^ Introducing GO into a multilayered MgAl‐LDH coating on an AZ31 Mg alloy may support the nucleation and in situ growth of MgAl‐LDH, thus forming a uniform and dense multilayer coating.^[^ ^29^, ^106^ ^]^ The synergistic effect of these materials impedes the diffusion of corrosive fluid, hindering its absorption and increasing impedance by more than tenfold. Second, doping with hydrophobic nanoparticles to prepare superhydrophobic coatings on the Mg alloy surface increases corrosion resistance.^[^ ^107^, ^108^ ^]^ SiO_2_‐F NPs dispersed uniformly in PDA coating may form a stable superhydrophobic surface with a WCA of 155° and thereby prevent the infiltration of inorganic ions such as Cl^−^, thus decreasing the corrosion current by an order of magnitude (8.084 × 10^−6^ A cm^−2^) compared with undoped coated samples (1.218 × 10^−5^ A cm^−2^).^[^ ^109^ ^]^ Third, impregnating NPs that have small particle sizes (such as nBG, ZnO NPs, and Ce‐HA NPs)^[^ ^26^, ^97^, ^104^ ^]^ on the surface of organic fibers may increase fiber diameter, improve surface chemistry, and reduce fiber gaps, thereby impeding the deposition and penetration of corrosive ions. For example, nBG increased the diameter of PCL nanofibers used to coat an AZ91 Mg alloy surface and thereby enhanced the interception of corrosive products and ions. Moreover, the precipitation of hydroxyapatite (the dissolved product of nBG) and Mg(OH)_2_ (the dissolved product of Mg) between nanofibers may further reduce the degradation rate of the underlying substrate, as reflected by a threefold reduction of the corrosion current and a nearly 100‐fold decrease of the degradation rate.^[^ ^104^ ^]^

The uniform distribution of nanomaterials and their bonding with other components of composite coatings increase compactness and reduce surface defects (including pores and cracks), thus regulating the degradation rate by decreasing porosity (Figure 3b). Two schemes are used for most applications. The first is the addition of NPs rich in —OH/—COOH groups (such as GO, SiO_2_ NPs) into an osteogenic Ca‐P coating that is prone to cracking.^[^ ^110^ ^]^ These nanoparticles bind to Ca^2+^ through valence bonds or electrostatic attraction to reduce surface cracking and delamination.^[^ ^111^ ^]^ For example, on an HA/GO composite nanocoating of an AZ31 Mg alloy surface, OH— and COO— groups of GO reacted with Ca^2+^ on HA to enhance the in situ nucleation and growth of HA.^[^ ^105^ ^]^ Meanwhile, the crack‐bridging mechanism of GO prevented crack propagation and increased the fracture toughness of the HA coating, thus reducing the stripping rate and increasing charge transfer resistance to decrease the degradation rate of Mg alloy.^[^ ^112^, ^113^ ^]^ The second scheme is to add bioactive NPs (such as nHA, Fe_3_O_4_ NPs, and ZnO NPs) into polymer coatings to regulate porosity, enhance stability, and delay the degradation of the composite nanocoating.^[^ ^74^ ^]^ Uniformly dispersed ZnO NPs in PLA coating decreased pore size from 5.5 ± 0.65 to 3.1 ± 0.25 µm, which reduced the penetration of corrosive ions and improved the degradation rate of the composite nanocoating from 3.779 to 5.199 × 10^−4^ mm year^−1^. The improved degradation rate of the coating, combined with osteogenic and antibacterial activities of ZnO NPs, may be broaden the applications of such coatings to include load‐bearing implants in the treatment of complex and nonunion fractures.

Furthermore, nanomaterials may enhance the adhesion between coating and substrate, thus preventing delamination and improving corrosion resistance (Figure 3c). Pre‐preparing nanocoatings by electrochemical conversion [such as N‐doped graphene quantum dots (N‐GQDs)]^[^ ^114^ ^]^ or by acid/alkali treatment followed by polymer coating to establish in‐situ nano‐MgF_2_/Mg(OH)_2_ coating could ensure stable corrosion resistance because the nanomaterials may form covalent bonds with polymers and atomic bonds with the Mg alloy substrate. Polymethyltrimethoxysilane (PMTMS) coating is prone to expansion and flaking when covering Mg alloys as a physical barrier.^[^ ^115^ ^]^ The functional groups of N‐GQDs were used to form chemical bonds between Si‐O‐N/Si‐O‐C and PMTMS. An AZ91 Mg alloy was then chemically bonded to the PMTMS coating, resulting in strong interface adhesion, which enabled the anchoring of the coating onto the substrate after 194 h of soaking. Corrosion resistance was thereby increased sixfold over that of uncoated Mg alloy).^[^ ^114^ ^]^

In addition, polymer coatings that are easily degraded by surface erosion [such as polyanhydrides and poly(orthoesters)] are prone to develop gaps in the junction with the substrate that weaken corrosion resistance.^[^ ^116^ ^]^ Such coatings exhibited gaps within 30 days in a dynamic simulated body fluid test (shear stress of about 0.68 Pa).^[^ ^117^ ^]^ In contrast, an alkali pretreated nano‐Mg(OH)_2_ layer interlocked with poly(1,3‐trimethylene carbonate) because similar polarity increased the contact area and adhesion strength between coating and substrate. This prevented rapid local corrosion induced by gaps between outer and inner layers. The coating remained tightly attached after 12 weeks of implantation in a rat femoral defect model, and substrate volume loss was only 14.8%.^[^ ^33^ ^]^

The development of self‐healing composite nanocoatings to improve corrosion resistance has attracted increasing attention (Figure 3d). Commonly used nanomaterials that inhibit corrosion include CeO_2_ NPs and F‐SiO_2_ NPs.^[^ ^15^, ^18^ ^]^ When a coating is damaged, these nanoparticles react with the released Mg^2+^ to form a protective film for passivation or to deposit products that cover the Mg alloy, thus preventing further corrosion.^[^ ^118^ ^]^ For example, in the composite coating prepared by blending CeO_2_ NPs, CS, and gelatin on orthopedic Mg alloy, CeO_2_ formed protective films on corroded sites, was reduced to Ce_2_O_3_, and attached to OH‐/H_2_O moieties to form an insoluble hydroxylated/hydrated Ce_2_O_3_ framework, thus filling the corrosion pits of AZ31 Mg alloy.^[^ ^15^, ^119^ ^]^ In addition, F‐SiO_2_ NPs released F^−^ to react with Mg^2+^ and form a precipitation layer to close micro‐defects caused by corrosive fluid infiltration.^[^ ^18^ ^]^ Other nanomaterials such as halloysite nanotubes^[^ ^120^ ^]^ and LDH^[^ ^121^ ^]^ can be used as microcontainers to carry corrosion inhibitors such as 8‐hydroxyquinoline and phytic acid,^[^ ^21^, ^122^, ^123^ ^]^ which have been studied in self‐healing nanocoatings for industrial Mg alloys. However, the development of self‐healing coatings for biomedical implants is still in its infancy, and may emerge among the leading research priorities if the safety of corrosion inhibitors can be improved.

### 3.3 Activating Cell Signaling Pathways to Promote Healing

#### 3.3.1 Osteogenic Signaling Pathways

**Figure 4:**
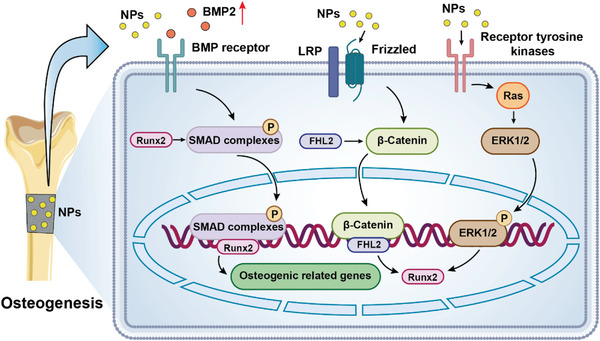
Nanoparticles in composite coatings promote osteoblast differentiation by activation of osteogenic signaling pathways. BMP2, bone morphogenetic protein 2; LRP, low density lipoprotein receptor‐related protein; NPs, nanoparticles.

In addition to promoting osteogenesis by accelerating apatite formation and deposition,^[^ ^36^, ^104^ ^]^ nanomaterials may upregulate osteogenic signaling pathways to promote cell proliferation, differentiation, and secretion of mineralized matrix.^[^ ^36^, ^83^, ^124^, ^125^, ^126^ ^]^ Composite nanocoatings of Mg alloy surfaces may act on BMP/SMAD,^[^ ^127^ ^]^ Wnt/*β*‐Catenin,^[^ ^128^ ^]^ ERK/MAPK,^[^ ^124^, ^129^ ^]^ and other signaling pathways related to osteogenic differentiation.^[^ ^130^ ^]^ These pathways regulate the key transcription factor Runx2 and the expression of major downstream osteogenic differentiation factors and genes (such as osterix, *ALP*, *OCN*, *COL‐1*, and *OPN*) to facilitate bone regeneration (**Figure** **4**). NPs based on calcium and phosphorus (e.g., nHA and nBG)^[^ ^131^, ^132^ ^]^ stimulated both BMP and Wnt pathways, which promoted the binding of Runx2 and phosphorylated SMAD complex to enhance expressions of osterix and *OCN*. These NPs also increased *β*‐catenin and FHL2 transcription that subsequently upregulated Runx2 synthesis, thus raising alkaline phosphatase expression. For example, a coating comprised of PCL, pluronic copolymers, and nHA applied to an AZ31 Mg mesh activated the BMP signaling pathway and downstream gene expression of MG63 cells 2‐5‐fold higher than a non‐composite coating. Furthermore, the coating promoted collagen secretion and extracellular mineralization, thereby accelerating new bone formation in long and difficult‐to‐heal segmental bone defects.^[^ ^133^, ^134^ ^]^ In addition, nanomaterials containing Li^+^,^[^ ^135^ ^]^ Sr^2+^,^[^ ^136^ ^]^ and F^−[^ ^137^ ^]^ also stimulate BMP and Wnt pathways, while graphene nanosheets,^[^ ^129^ ^]^ Mg‐Al LDH,^[^ ^138^ ^]^ and iron oxide NPs^[^ ^139^ ^]^ activate MAPK signaling pathways.^[^ ^140^ ^]^ Composite nanocoatings utilizing these NPs may stimulate osteoblastic proliferation and differentiation.^[^ ^124^ ^]^

Immune‐mediated activation of the NF‐*κ*B pathway drives osteoclast differentiation, osteolysis, and inflammation. The addition of nanoparticles that upregulate OPG, inhibit RANKL‐RANK interactions, or increase the OPG/RANKL ratio may regulate bone homeostasis by inhibiting the NF‐*κ*B‐mediated expression of osteoclast differentiation genes.^[^ ^26^, ^80^ ^]^ The addition of Mg‐Al LDH to a MgO ceramic coating significantly diminished proinflammatory M1 macrophage activation and downregulated RANKL expression and nuclear transfer of p65 to suppress NF‐*κ*B signaling and osteoclast differentiation. Consequently, the coating generated a less inflammatory microenvironment (with a higher proportion of M2 macrophages) after in‐vivo implantation, thus reducing bone resorption and promoting osseointegration.^[^ ^83^, ^141^ ^]^

Novel nanoparticles activate osteogenic pathways to promote bone healing. Tantalum nanoparticles promote osteoblast differentiation through BMP and Wnt signaling pathways.^[^ ^142^, ^143^ ^]^ Au NPs^[^ ^144^, ^145^ ^]^ activate Runx2 through the YAP/TAZ signaling pathway to upregulate osteoblastic differentiation of BMSCs.^[^ ^146^, ^147^, ^148^ ^]^ Manganese NPs modulate bone metabolism via parathyroid hormone signaling pathways,^[^ ^149^, ^150^ ^]^ and regulate ROS by their antioxidant properties to affect osteogenic signaling pathways (e.g., MAPK, Wnt, NF‐*κ*B).^[^ ^151^ ^]^ Cobalt‐doped metal–organic frameworks and carbon nanotubes release low doses (≈1 ppm) of Co^2+^ to regulate osteoimmunology through the NF‐*κ*B pathway to enable minimally invasive treatment of neoplastic bone defects.^[^ ^152^, ^153^, ^154^ ^]^ Regrettably, these nanoparticles have not been applied to Mg alloy coatings. Consequently, their potential utility as coatings for Mg alloy implants remains a promising topic for future research.

#### 3.3.2 Angiogenic Signaling Pathways

**Figure 5:**
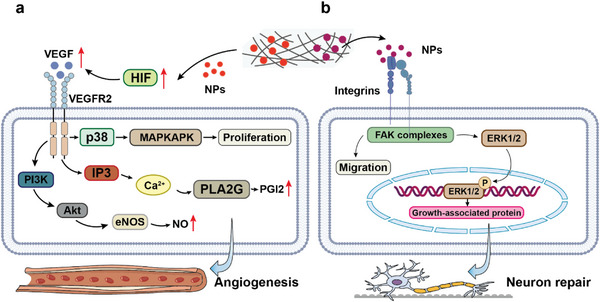
Nanoparticles in composite coatings promote tissue healing through pathways associated with a) angiogenesis and b) nerve repair. HIF, hypoxia inducible factor; NPs, nanoparticles; VEGF, vascular endothelial growth factor.

Whether to prevent stent thrombosis or to improve tissue perfusion, the promotion of Mg alloy stent endothelialization is imperative. Nanomaterials such as GO^[^ ^30^ ^]^ and SiO_2_ NPs^[^ ^22^ ^]^ increase vascular endothelial growth factor (VEGF) expression mainly through the upregulation of hypoxia inducible factor (HIF), which binds to the VEGF receptor 2 to activate angiogenic signaling pathways. These include the MAPK pathway that promotes EC proliferation; the downstream calcium pathway that produces prostacyclin to induce vasodilation and attenuate platelet aggregation; the PI3K‐Akt pathway that upregulates NO expression to promote EC migration, inhibit vascular smooth muscle contraction and growth and attenuate platelet aggregation (**Figure** **5**a).^[^ ^155^ ^]^ Hydrophobic SiO_2_ NPs dispersed uniformly in a poly(l‐lactide) matrix to form a composite nanocoating on the Mg alloy surface of vascular stents release silicon ions slowly to inactivate prolyl hydroxylase and activate HIF‐1, thus promoting stent endothelialization through the VEGF signaling pathway.^[^ ^22^ ^]^

In addition, early angiogenesis during bone healing facilitates the delivery of nutrients and osteogenic cells. Tissue perfusion is imperative for bone repair. The primary ossification center in endochondral ossification is derived from osteoblasts from blood vessels and perichondrium; the osteogenic differentiation of osteoprogenitor cells in intramembranous ossification (e.g., that occurs in the skull) also depends on vascularized periosteum.^[^ ^156^ ^]^ The healing of fractures accompanied by periosteal and perichondrial dissection is delayed due to inadequate vascularization.^[^ ^157^ ^]^ Nano‐mineralized collagen (nHAC) mixed with polymethyl methacrylate and CS and deposited on an Mg‐Ca alloy surface induced VEGF secretion and consequent EC proliferation and migration.^[^ ^25^, ^158^ ^]^ This coating facilitated angiogenesis and osteogenesis within four weeks of implantation in a rabbit calvarial defect model, while coatings without nHAC formed uneven nodular bone until 24 weeks after surgery. In addition to activating the VEGF signaling pathway, Au NPs^[^ ^159^ ^]^ or NPs containing Zn^2+^ or Sr^2+[^ ^160^ ^]^ upregulate Ang I through the Ang‐Tie signaling pathway to mediate EC migration, adhesion, and proliferation; thus, stimulating early angiogenesis. The profound pro‐angiogenic activity of these nanomaterials may provide directions for future studies.

#### 3.3.3 Neuroregenerative Signaling Pathway

Mg^2+^ benefits neuroregeneration and functional recovery. Consequently, Mg alloy implants can serve as nerve guide conduits or scaffolds to guide neural tissue regeneration.^[^ ^85^, ^88^ ^]^ Nanomaterials with electrical conductivity and neuroinduction properties (such as carbon nanotubes and black phosphorus)^[^ ^16^, ^90^ ^]^ may be incorporated into composite nanocoatings. These NPs upregulate GAP‐43 expression through integrin/FAK and downstream MAPK signaling pathways to facilitate axonal regeneration and conduction circuit remodeling (**Figure** 5b).^[^ ^161^, ^162^ ^]^ Because the reparative capacity of the peripheral nervous system is limited, enhanced Schwann cell proliferation and migration to the axonal terminus of injured neurons and subsequent remyelination are essential for nerve repair. Composite coatings comprised of tubular nanomaterials [such as carbon nanotubes (CNTs) and PCL nanofibers] may promote neuronal adhesion, differentiation, and axonal extension through integrin‐mediated interactions between the implant and the neuronal cell membrane. For example, soluble CNTs from a CNTs‐CaP/CS‐coated AZ91D Mg alloy upregulated p‐FAK through the integrin pathway, and then activated MAPK signaling to increase GAP‐43 expression to promote axon elongation, neurite regeneration, and neuronal repair in the rat dorsal root ganglion.^[^ ^16^, ^161^ ^]^ Collagen‐functionalized PCL nanofibers that carry antioxidant lignin nanoparticles activated *α*2*β*1 integrin that recognized type I collagen, stimulated downstream tyrosine kinase FAK, and promoted directional elongation of neurites and Schwann cell migration to facilitate myelination. In an embryonic chicken model, these nanofibers extended the growth of dorsal root ganglia beyond 1800 µm in only 6 d.^[^ ^163^, ^164^, ^165^, ^166^, ^167^ ^]^ NPs may protect neurons and glial cells from oxidative stress^[^ ^168^ ^]^ and expedite the regeneration of central and peripheral nervous systems.^[^ ^169^, ^170^, ^171^ ^]^ Therefore, the addition of these NPs to Mg alloy surfaces may advance the field of neuroregeneration.

### 3.4 Role as Drug Carriers

**Figure 6:**
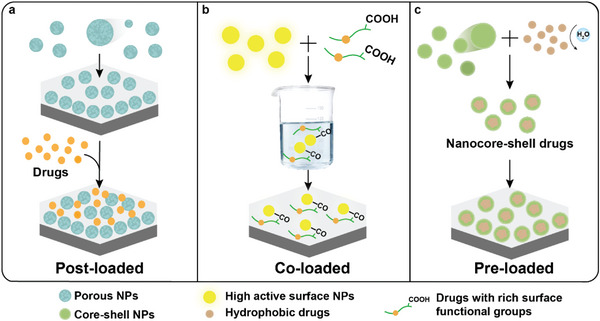
Composite nanocoatings may serve as drug carriers. a) Post‐loaded: small‐molecule drugs are adsorbed on the coating containing porous nanomaterials. b) Co‐loaded: drugs with rich surface functional groups are combined with nanomaterials with highly active surfaces. c) Pre‐loaded: nanomaterials with core–shell structure preloaded with hydrophobic drugs.

Cytological and molecular responses at the bone–implant interface are critical for successful implantation. Modifying the implant surface to load drugs and regulate the interface response may address implant‐related complications such as aseptic loosening and infection.^[^ ^172^, ^173^ ^]^ An implant‐based strategy may pose fewer biological barriers and offer more immediate results than systemic antibiotic or anti‐inflammatory therapies. Nanomaterials may be loaded with drugs and bioactive molecules. Therapeutic payloads may include pro‐osteogenic agents such as bone morphogenic protein and simvastatin;^[^ ^174^, ^175^ ^]^ pro‐endothelialization drugs such as propranolol;^[^ ^176^ ^]^ anti‐inflammatory drugs including ibuprofen and diclofenac;^[^ ^177^, ^178^ ^]^ and antibiotics such as ciprofloxacin and gentamicin^[^ ^179^ ^]^ to accelerate fracture union promote, reduce local inflammation, and prevent infection. The high specific surface areas and adsorption capacities of nanomaterials enable drug stabilization and sustained release through three mechanisms.^[^ ^180^ ^]^ The first is the preparation of porous nanocarriers on the substrate surface to load small‐molecule drugs and achieve high‐dose drug‐loading and nanosized drug delivery (**Figure** **6**a). The second approach is the superposition or doping of medications [such as bone morphogenetic protein 2 (BMP‐2), diclofenac, and propranolol]^[^ ^24^, ^178^, ^181^ ^]^ onto NPs during the synthesis of composite nanocoatings. These NPs may enable controlled drug release as the coatings gradually degrade (**Figure** 6b). Third, core–shell structure NPs with stable shells can protect hydrophobic or rapidly metabolized drugs (including curcumin, simvastatin, and gentamicin)^[^ ^18^, ^175^, ^182^ ^]^ that are pre‐loaded into the core to realize high‐efficiency and slow‐release delivery (**Figure** 6c).^[^ ^180^ ^]^

Nanomaterials with porous structures (including SiO_2_ NPs, nHA, and polymer nanofibers)^[^ ^26^, ^177^, ^179^, ^182^ ^]^ may load small‐molecule drugs by physical adsorption or electrostatic interaction to generate composite nanocoatings on Mg alloy surfaces and thereby improve anti‐inflammatory and antibacterial activities. An in vitro drug release study of ibuprofen adsorbed on the surface of PCL nanocomposite fibers modified with ZnO NPs showed that ibuprofen release was rapid during the first two days, but slowed in medium term (days 2–7) with the expansion of PCL. The ZnO NPs increased the adhesion of ibuprofen to PCL nanofibers, enabling a sustained release during the controllable decomposition of nanocomposite fibers over a period of 22 d.^[^ ^177^ ^]^ Second, drugs with abundant functional groups may be blended or superposed on nanomaterials with high surface activity (e.g., LDH, nHA, GO)^[^ ^24^, ^181^, ^183^ ^]^ to form composite nanocoatings with greater drug loading capacity compared to the direct deposition of drugs on the substrate surface. The deposition of a BMP‐2, PDA, and nHA composite on an AZ31 Mg alloy surface resulted in coupling of the catechol/quinone groups of PDA to BMP‐2, enabling a continuous release of BMP‐2 (35% release in 5 d and 67% in 28 d). In contrast to the 90% release of BMP‐2 within 5 d from coatings prepared by direct dipping, a coating incorporating nHA enabled sustained release over a 12‐week duration to continuously promote osteoblast differentiation and bone repair in a rabbit femoral defect model.^[^ ^24^ ^]^ Ciprofloxacin with —COOH groups assembled alternately with poly(ethyleneimine) (PEI) by electrostatic interaction was doped onto nHA to form a composite nanocoating. The interaction between —COOH and Ca^2+^ and the barrier effect of PEI prolonged drug elution, which enabled continuous ciprofloxacin release at an effective concentration during the 25‐day coating degradation.^[^ ^179^ ^]^ In addition, nanomaterials with drug‐carrying structures [such as mesoporous SiO_2_ NPs and gelatin nanospheres (GNs)]^[^ ^18^, ^175^ ^]^ in composite nanocoatings provide stable shells that may encapsulate hydrophobic drug cores. These particles can be adapted to adjust drug dose and loading sites and to create a diffusion barrier for controlled drug release. Simvastatin, which promotes osteoblast differentiation and extracellular matrix mineralization, was preloaded into GNs in chitosan to modify a WE43 Mg alloy. Compared to physical adsorption on the PCL/nHA coating that released more than 80% of simvastatin in 4 d, the addition of GNs not only eliminated the initial burst release (higher than the cytotoxicity threshold of 0.05 µg mL^−1^) that may inhibit cell proliferation, but also achieved a sustained release over 28 d.^[^ ^184^ ^]^ To achieve an ideal antibacterial drug delivery that features an initial burst to provide an effective loading dose followed by sustained continuous release, silica‐gentamicin (Si‐G) nanoparticles and tetraethylorthosilicate‐methyltriethoxysilane‐bioactive glass (TMB) formed a double coating (TMB/Si‐G/TMB/Si‐G) that exerted a double time‐dependent gentamicin release.^[^ ^182^ ^]^ During the brief critical period after Mg alloy implantation (3 h), a high initial dose of gentamicin was released from the first Si‐G layer to inhibit early bacterial adhesion, while the TMB interlayer impeded the diffusion of the second Si‐G layer and extended the overall release time, thus providing sustained antibacterial activity to enhance bone healing at an infected site.

## 4 Design Strategies of Composite Nanocoatings on Mg Alloys

### 4.1 Distribution of Nanomaterials Affects the Function of Composite Nanocoating

**Figure 7:**
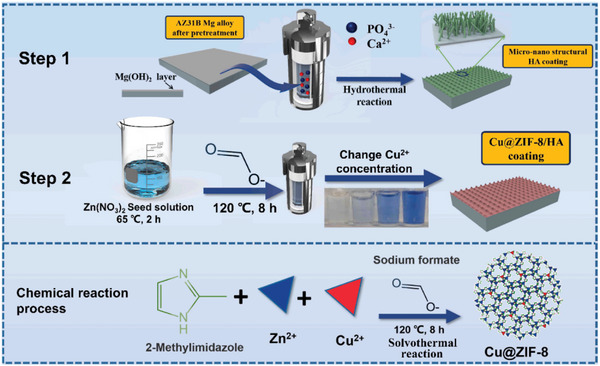
Preparation process and reaction mechanism of in situ growth of Cu@ZIF‐8/HA composite coatings on AZ31B Mg alloy via a two‐step approach of hydrothermal treatment and seeded solvothermal method. Reproduced with permission.^[^ ^27^ ^]^ Copyright 2022, Elsevier.

The function of incorporated nanomaterials is closely related to their distribution and location on the implant. LBL multilayer coatings made by assembly/superposition offer the advantages of highly dispersed, uniformly deposited nanoparticles that afford controllable thickness (**Figure** **7**).^[^ ^185^ ^]^ Interfacial adhesion is improved by positioning at the base layer;^[^ ^186^ ^]^ biological effects are optimized at the outer layer;^[^ ^30^ ^]^ and a relatively stable release curve may be maintained when alternating cycle assembly.^[^ ^187^ ^]^ In addition, increased NP content reduces the porosity of the coating and improves the physical barrier effect.^[^ ^12^ ^]^ However, with increased doping, the agglomeration of nanomaterials with high surface activity can cause uneven stress. Aggregates may serve as crack nucleation points to break the integrity of the coating and degrade its performance.^[^ ^12^, ^17^ ^]^ For example, doping 0.005, 0.05, 0.5, and 1 (wt%) COOH‐functionalized graphene nanosheets (COOH‐GNP) into TEOS/MTES mixtures to prepare sol‐gel composite nanocoatings of an AZ31 Mg alloy used in orthopedic devices disclosed that the protective behavior of the coating was directly related to GNP content up to 0.05 wt% (corrosion current was two orders of magnitude lower than that of the uncoated sample). However, when the GNP content exceeded 0.05 wt%, GNPs formed aggregates in the coating, which became internal stress concentration points that generated cracks and irregular coating morphology, thus degrading protective properties.^[^ ^17^ ^]^

### 4.2 Service Environment Determines the Optimal Ratio of Nanoparticles in Composite Coating

The varied biological effects of multifunctional composite nanocoatings must be balanced in proportion to their intended use. Consequently, the ratios of NPs with specific biological effects should be adapted. Antibacterial activity, biocompatibility, corrosion resistance, and osteogenic activity must be considered. For example, antibacterial nanoparticles used on implants to prevent infections of open fractures may raise the risks of cytotoxicity. These nanomaterials (such as TiO_2_ or Ag NPs)^[^ ^14^ ^]^ may cause oxidative stress in both bacterial and host cells simultaneously, leading to the unintended consequence of host cell apoptosis. Consequently, antibacterial potency and biocompatibility must be balanced.^[^ ^73^ ^]^ Furthermore, the corrosion resistance and biological properties of nanomaterials are related. A high degradation rate of a coating enables the rapid release of bioactive nanoparticles, which could promote a local healing microenvironment but reduce corrosion resistance, resulting in the rapid decline of substrate's mechanical performance. However, although low degradability is a prerequisite for the preservation of mechanical properties, the consequent slow release of bioactive nanoparticles may confound the antibacterial or osteogenic activities of the implant. Not surprisingly, corrosion resistance and antibacterial activity are difficult to balance. For example, evaluation of a chitosan coating that incorporated nBG and three different concentrations [1, 3, 5 (wt%)] of Fe_3_O_4_ NPs demonstrated that the sample with only 1 wt% Fe_3_O_4_ NPs achieved the optimal corrosion resistance.^[^ ^19^ ^]^ Though the samples with 3 and 5 (wt%) Fe_3_O_4_ NPs exerted a more potent short‐term antibacterial effect, the rapid degradation rate led to the premature release and consumption of Fe_3_O_4_ NPs, which reduced long‐term antibacterial activity. Therefore, the further exploration of nanoparticles with higher biological effects at lower concentrations is essential to optimize the properties of composite nanocoatings. Moreover, current studies have rarely focused on the effects of varied concentrations of doped nanoparticles on performance, and typically address only one component of composite nanocoatings. Insufficient attention has been paid to adapting the ratios of different components. This topic clearly requires further study.^[^ ^19^ ^]^

### 4.3 Practical Preparation Schemes and Technology Are Critical to Realize the Clinical Benefits of Composite Nanocoatings

**Figure 8:**
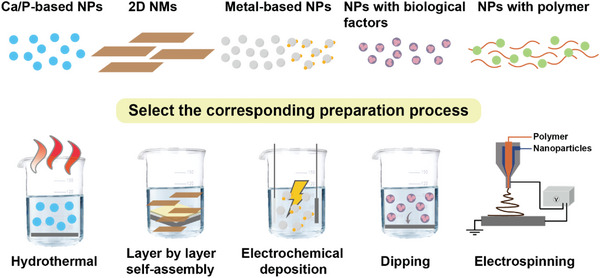
Schematic diagram of different nanomaterials and preparation schemes that may affect the properties of composite nanocoatings. 2D, two‐dimension; NMs, nanomaterials; NPs, nanoparticles.

Variations in preparation schemes and manufacturing parameters affect the microstructure and bioefficacy of coatings. Practical preparation schemes and processes are essential to realize the intended benefits of the composite coating (**Figure** **8**). Some scholars have summarized specific schemes of Mg alloy modification: for example, simple thin chemical conversion coatings have strong binding forces but insufficient strength; electrochemical coatings are porous; physical deposition coatings have high corrosion resistance but weak binding forces.^[^ ^7^, ^12^, ^33^, ^34^, ^36^, ^68^, ^188^ ^]^ Hence, designing realistic preparation schemes and selecting optimal processes to manufacture particular nanomaterials that are adapted to specific service environments will confer optimal coating characteristics and improve implant success rates.

Particular clinical applications require corresponding coating preparation schemes. Bone implants require compact coatings with robust mechanical performance. Consequently, nanomaterials (such as nHA, GO, Ag, and ZnO NPs) are usually mixed into the electrolyte to prepare composite nanocoatings with controllable thickness and uniform particle distribution by electrochemical deposition. The coatings of Mg alloy cardiovascular stents are typically prepared by superposition methods such as spin‐coating or LBL self‐assembly, which may precisely control both the lumenal components that contact the bloodstream and the components of surrounding layers.^[^ ^30^ ^]^ In addition, different nanomaterials require corresponding preparation processes to exert their intrinsic properties. NPs with calcium and phosphorus as main components (e.g., nHA, nBG, etc.) have stable osteogenic properties. Composite coatings containing these NPs are usually prepared by thermal conversion, which is a cost‐effective and environmentally friendly technique, but may result in thin and uneven coatings. In recent years, electrochemical deposition has emerged as the preferred method to prepare nanocoatings on Mg alloy surfaces. This technique controls porosity and thickness by adjusting parameters, thereby balancing the degradation rate and osteogenic effect.^[^ ^36^ ^]^ 2D nanomaterials with abundant surface charge, such as GO and LDH, are usually doped by electrodeposition or self‐assembly to prepare multilayer anti‐corrosion films. This method regulates nanomaterial distribution to obtain stable and uniform dispersion that improves the protective performance of composite nanocoatings.^[^ ^189^ ^]^ To synthesize composite nanocoatings that require doping with biological factors or protein components such as BMP‐2, heparin, or bovine serum albumin, a mixed solution with nanoparticles may be prepared by spin‐coating, thereby forming a uniform and dense coating without inactivating the attached biological factors.^[^ ^24^, ^186^, ^190^ ^]^ For composite coatings containing superpositing polymers, nanocoatings are generally prepared by LBL self‐assembly or chemical treatment, followed by polymer dipping to obtain a corrosion‐resistant coating with a close chemical bond to the substrate.^[^ ^31^, ^33^, ^114^ ^]^ Polymer nanofibers such as PCL/PLA prepared by electrostatic spinning can directly compound metal or metal‐oxide nanoparticles. This method adjusts the diameter and aperture of the fibers, thus promoting bioefficacy and regulating the degradation of the composite nanocoating, recon without increasing process complexity.^[^ ^97^ ^]^

### 4.4 Degradation Products of Nanocoatings Should Facilitate Tissue Healing and Be Readily Metabolized

**Figure 9:**
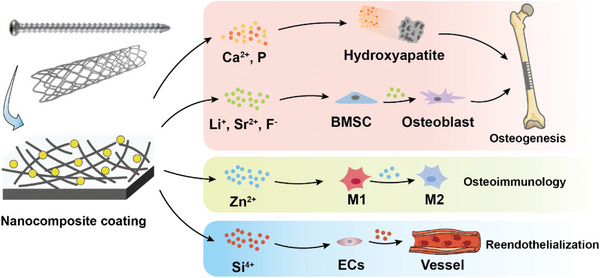
Degradation products of composite nanocoatings should benefit tissue healing and be readily metabolized. BMSC, bone marrow mesenchymal stem cell; ECs, endothelial cells.

The degradation products of nanocoatings should be biocompatible and readily metabolized. Degradation of the composite layer will change the surrounding microenvironment (e.g., alter pH and ion concentrations), which should be considered during the design of nanocoatings. Degradation products should neither incite inflammation nor predispose to implant‐associated infections, and should promote cell adhesion and differentiation through the release of degradation products that enhance osseointegration and angiogenesis (**Figure** **9**).^[^ ^191^, ^192^ ^]^ Calcium and phosphorus ions released by the degradation of nanocoatings containing Ca/P components can supplement the mineral reservoir to provide substrates for the deposition of hydroxyapatite, thus promoting bone regeneration.^[^ ^193^ ^]^ Released Li^+^, Sr^2+^, and F^−^ activate signaling pathways related to BMSC osteogenic differentiation and may promote bone integration.^[^ ^135^, ^136^, ^137^ ^]^ Zn^2+^ is a key regulator of the NF‐*κ*B pathway of macrophages in osteoimmunology,^[^ ^26^, ^27^, ^194^ ^]^ while silicon ion upregulates HIF to activate the VEGF signaling pathway of ECs to promote vascular regeneration and reendothelialization.^[^ ^22^ ^]^ Based on the mechanism described above, composite nanocoatings containing nHA/nBG/SrCO_3_/ZnO/SiO_2_ nanoparticles may be designed to release ions that promote healing.

In addition, second‐phase nanomaterials can partially offset poor biocompatibilities of primary nanocoat components. Acidic degradation products of polymers such as PLA or PLGA will reduce the pH of adjacent tissues; consequently, second‐phase alkaline nanomaterials may compensate and thereby prevent aseptic inflammation. For example, dispersing nHA into a PLGA matrix (30 wt% nHA and 70 wt% PLGA) may neutralize acid‐base degradation products, minimizing local pH changes and improving the bioactivity of implants for osseointegration. However, carbon‐based nanomaterials such as GO and noble metal nanoparticles such as Au/Ag may produce ROS and cause oxidative stress after endocytosis;^[^ ^195^ ^]^ rare‐earth nanomaterials are difficult to degrade; and nanoparticles excreted by cells may enter the bloodstream and injure distant tissues and organs.^[^ ^196^ ^]^ Research of in‐vivo metabolism of nanomaterials is still in its infancy. Consequently, the toxicity of coating degradation products to local microenvironments and distant organs is incompletely understood. Therefore, a consideration of metabolism and safe dosing is imperative during the design of composite nanocoatings. Attention must be paid to potential cytotoxicity while improving the intended therapeutic effects of implants to realize real clinical benefits.

## 5 Summary and Future Prospects

Mg alloy was proposed for biomedical applications as early as 1878. However, its rapid biodegradation limits its clinical applications.^[^ ^197^ ^]^ Composite nanocoatings optimize the degradation rate of Mg alloy to meet mechanical performance requirements and improve osteogenesis and angiogenesis. Coatings may also produce a peri‐implant microenvironment conducive to tissue repair and healing by preventing thrombosis and stenosis of vascular stents, and through immunomodulatory and antibacterial activity. The underlying mechanism of composite nanocoating with enhanced biological properties may include the following aspects: 1) altered physical (permeability) and chemical (bonding or covalent bonding) properties of nanomaterials that regulate the degradation rate of Mg alloy; 2) promotion of cell adhesion and subsequent proliferation and differentiation; 3) biodegradability and subsequent release of nanoparticles into the implant microenvironment, thus regulating signaling pathways related to osteogenesis, angiogenesis, and neuroregeneration; and 4) transport and controlled release of therapeutic payloads. Therefore, the proportion and distribution of nanomaterials, either by mixing in a single layer or superimposing into multilayers, should be determined according to the needs of the implant microenvironment. Balancing the component ratios for specific clinical applications is necessary to attain therapeutic goals. Furthermore, the preparation scheme and fabrication process should be designed to meet application requirements and should consider the unique and potentially complementary characteristics of particular nanomaterials, thus designing fully multifunctional composite nanocoatings and improving the success rate of Mg alloy implants.

In conclusion, the development and application of composite nanocoatings will significantly broaden the application range and improve the efficiency of Mg alloy implants. The most promising directions for future research in this field lie in the following three aspects. First, enriching the type of mixed/doped nanomaterials is still the primary direction of constructing and developing composite nanocoatings, especially the combination of nanoparticles with synergistic and complementary effects. However, the impact of biosafety after the introduction of multiple kinds of nanoparticles should also be taken into account. Second, as nanomaterials with excellent biological activities are constantly being discovered, the attemptation to combine new NPs into composite coatings may significantly improve the scope of clinical applications of Mg alloys.^[^ ^198^ ^]^ For example, black phosphorus promotes stem cell differentiation, and could facilitate the application of Mg alloy scaffolds for neuroregeneration and neural tissue engineering.^[^ ^199^ ^]^ Nanozymes mimicking catalase activity (such as Pd, Mn‐based nanozymes) can reduce cellular oxidative stress, and their application in composite nanocoatings of Mg alloy cardiovascular stents improves their efficacy in the treatment of atherosclerosis.^[^ ^200^ ^]^ Composite nanocoatings that incorporate Ce‐based NPs that simulate peroxidase to induce apoptosis may facilitate the use of Mg alloy implants to repair bone defects that complicate tumor resection.^[^ ^201^, ^202^ ^]^ Third, though composite nanocoatings may greatly improve the utility of Mg alloys, obstacles to their commercialization and widespread application must be overcome. The service time of modified Mg alloys ranges from 4 to 12 weeks, which is below the ideal degradation time (more than three months) of orthopedic implants and vascular stents. Therefore, the further exploration of nanoparticles with high corrosion resistance still stands in the mainstream of future work.

## Conflict of Interest

The authors declare no conflict of interest.
